# FOXC2 augments tumor propagation and metastasis in osteosarcoma

**DOI:** 10.18632/oncotarget.11990

**Published:** 2016-09-13

**Authors:** Maricel C. Gozo, Dongyu Jia, Paul-Joseph Aspuria, Dong-Joo Cheon, Naoyuki Miura, Ann E. Walts, Beth Y. Karlan, Sandra Orsulic

**Affiliations:** ^1^ Women's Cancer Program at the Samuel Oschin Comprehensive Cancer Institute, Cedars-Sinai Medical Center, Los Angeles, CA, USA; ^2^ Graduate Program in Biomedical Science and Translational Medicine, Cedars-Sinai Medical Center, Los Angeles, CA, USA; ^3^ Department of Biochemistry, Hamamatsu University School of Medicine, Hamamatsu, Japan; ^4^ Department of Pathology and Laboratory Medicine, Cedars-Sinai Medical Center, Los Angeles, CA, USA; ^5^ Department of Obstetrics and Gynecology, David Geffen School of Medicine, University of California, Los Angeles, Los Angeles, CA, USA

**Keywords:** anoikis, forkhead box C2, osteosarcoma, metastasis, invasion

## Abstract

Osteosarcoma is a highly malignant tumor that contains a small subpopulation of tumor-propagating cells (also known as tumor-initiating cells) characterized by drug resistance and high metastatic potential. The molecular mechanism by which tumor-propagating cells promote tumor growth is poorly understood. Here, we report that the transcription factor forkhead box C2 (FOXC2) is frequently expressed in human osteosarcomas and is important in maintaining osteosarcoma cells in a stem-like state. In osteosarcoma cell lines, we show that anoikis conditions stimulate FOXC2 expression. Downregulation of FOXC2 decreases anchorage-independent growth and invasion *in vitro* and lung metastasis *in vivo*, while overexpression of FOXC2 increases tumor propagation *in vivo*. In osteosarcoma cell lines, we demonstrate that high levels of FOXC2 are associated with and required for the expression of osteosarcoma tumor-propagating cell markers. In FOXC2 knockdown cell lines, we show that CXCR4, a downstream target of FOXC2, can restore osteosarcoma cell invasiveness and metastasis to the lung.

## INTRODUCTION

Osteosarcomas are aggressive bone malignancies derived from osteoblast progenitor cells of mesenchymal origin [1]. These primary bone tumors, which frequently arise during period of rapid bone growth, most commonly affect children and young adults and are the second highest cause of cancer-related death in children and adolescents [2]. Micrometastases are detected in approximately 90% of patients during initial diagnosis. Despite advancements in neoadjuvant therapy and treatment, the survival rate has remained at 60% for the past decade [3]. It has been suggested that the lack of improvement in survival may be due to the inability to effectively target tumor-propagating cells in osteosarcoma [4]. Accumulating evidence shows that tumor-propagating cells originate from a small population of undifferentiated cancer cells with stem-like properties [5, 6]. Identifying molecular pathways that regulate the maintenance and expansion of tumor-propagating cells is critical to designing effective targeted therapy against these cells [7].

Conditions that generate and sustain tumor-propagating cells are not completely understood. It is thought that the signaling pathways involved in cellular differentiation are dysregulated within a tumor tissue environment, leading to cancer cells with stem-like properties [8]. Previous studies that focused on phenotypic characterization of tumor-propagating cells have used various cell surface markers known to induce stem-like and mesenchymal phenotypes and/or drug resistance, such as CD117, Stro-1, CXCR4 and ABCG2, and CD133 [10–12].

FOXC2 is a gene involved in epithelial to mesenchymal transition (EMT), a biological process acquired by cancer cells during cancer progression [13]. Overexpression of FOXC2 in an immortalized human mammary epithelial cell line expressing the *ras* oncogene (HMLER) resulted in a more mesenchymal stem-like state [14]. It has been shown that elevated FOXC2 expression is associated with increased metastasis in several cancer types, including breast, colorectal, nasopharingeal and esophageal [15–21]. In colorectal and esophageal cancer, FOXC2 expression correlated with poor prognosis and the loss of FOXC2 function decreased the invasive and migratory abilities of cancer cells *in vitro* [15, 17–19].

It is unknown if FOXC2 plays a role in non-epithelial malignancies where EMT is not essential for tumor progression. Because of the presence of FOXC2 in skeletal precursor cells [22] and involvement in osteogenic differentiation [23–26], we hypothesized that FOXC2 may play a role in a tumor type with altered osteogenic differentiation, such as osteosarcoma. In this study, we investigated the role of FOXC2 in osteosarcoma growth and metastasis. We demonstrate that FOXC2 plays a critical role in osteosarcoma pathogenesis by enhancing the ability of tumor-propagating cells to metastasize and propagate tumors.

## RESULTS

### FOXC2 is highly expressed in osteosarcoma primary tumors and lung metastases

**Figure 1:**
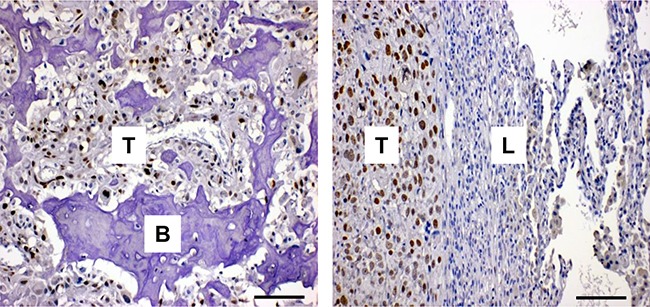
FOXC2 is expressed in human osteosarcoma Representative images of FOXC2 immunostaining in six primary osteosarcomas (left panel) and four osteosarcoma metastases to the lung (right panel). Hematoxylin was used as background staining. T, tumor; B, bone; L, lung. Scale bar, 100 μm.

Examination of the relative expression of FOXC2 in a diverse range of cancer cell lines in the Cancer Cell Line Encyclopedia (CCLE) expression profile database showed that FOXC2 mRNA levels were elevated in several cancer types, including osteosarcoma (data not shown). Additionally, FOXC2 mRNA was upregulated in osteosarcoma samples in comparison to normal bone (Supplementary Figure S1A) and in osteosarcoma lung metastases in comparison to primary tumors (Supplementary Figure S1B). We assessed the protein levels of FOXC2 in tumor tissue biopsies from patients diagnosed with Ewing's sarcoma, embryonal rhabdomyosarcoma, and osteosarcoma. While positive FOXC2 immunostaining was observed in 3 of 7 Ewing's sarcomas and 1 of 2 embryonal rhabdomyosarcomas, 9 of 10 examined osteosarcomas were positive for FOXC2 with 7 tumors exhibiting strong staining and the remaining 2 tumors exhibiting weak staining (Supplementary Table S1). Positive FOXC2 staining was observed in primary bone lesions as well as in lung metastases from osteosarcoma patients (Figure 1). The frequent detection of FOXC2 expression in human osteosarcomas suggests a possible involvement of FOXC2 in osteosarcoma pathology.

### Anoikis increases FOXC2 expression in osteosarcoma cell lines

**Figure 2:**
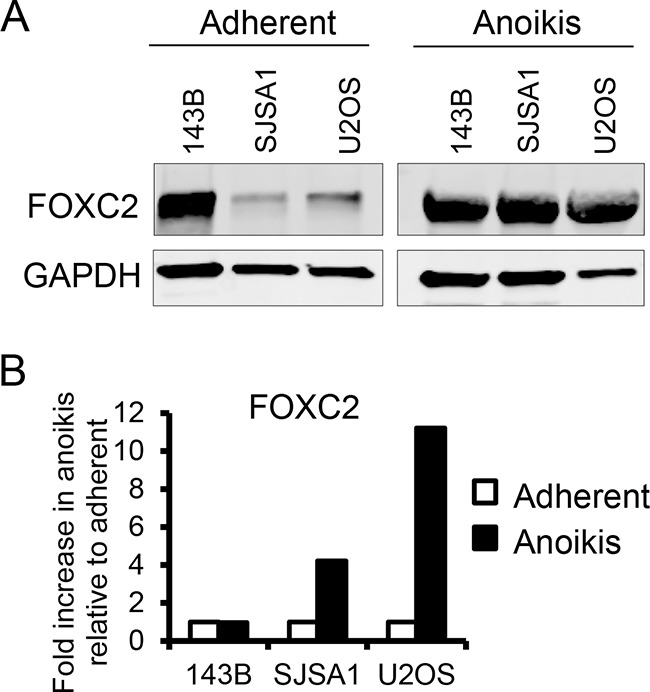
Anoikis increases FOXC2 levels Protein and mRNA levels of FOXC2 in osteosarcoma cell lines cultured in adherent and anoikis conditionswere assessed by **A.** Western blotting and **B.** qRT-PCR, respectively.

To assess the potential involvement of FOXC2 in the metastatic capability of osteosarcoma, we analyzed the effects of culture conditions in the osteosarcoma cell lines 143B (exhibits high endogenous levels of FOXC2) and in SJSA1 and U2OS (exhibit moderate endogenous levels of FOXC2) (Figure 2A). Metastatic dissemination requires the detachment of cells from the primary tumor and survival in anoikis conditions. To simulate anoikis, osteosarcoma cell lines were grown in non-adherent cell culture dishes. In comparison to adherent conditions, expression of the FOXC2 protein (Figure 2A) and mRNA (Figure 2B) were promoted in anoikis conditions in the SJSA1 and U2OS cell lines to levels comparable to the metastatic cell line 143B. These data indicate that survival in anoikis conditions may be aided by FOXC2 expression.

### Silencing of FOXC2 expression attenuates anchorage-independent growth and invasion capabilities of osteosarcoma cells *in vitro*

**Figure 3:**
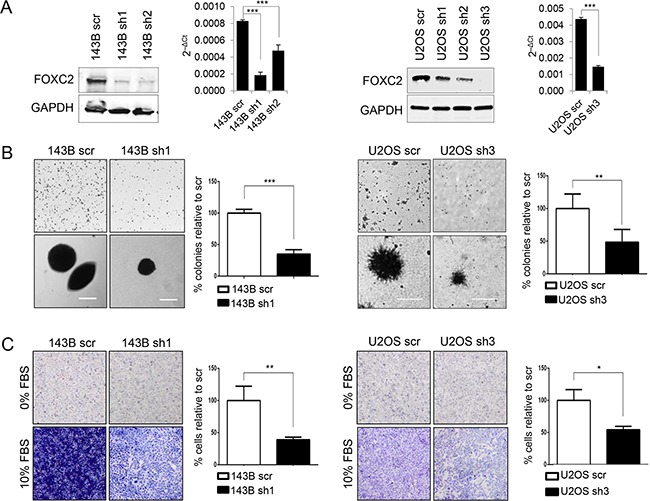
FOXC2 silencing diminishes anchorage-independent growth and metastasis **A.** Western blot and qRT-PCR analyses of FOXC2 expression in 143B and U2OS cell lines transduced with scrambled control shRNA (scr) or shRNAs specific for FOXC2 (sh1-sh3). **B.** Representative images and graphical quantifications of soft agar colonies formed by control cells and FOXC2 knockdown cells. Images at the bottom are enlarged to show the size of individual representative colonies. Scale bar, 100 μm. **C.** Representative images and graphical quantifications of 48 h Matrigel invasion by control cells and FOXC2 knockdown cells in the presence or absence of 10% FBS chemoattractant. *p<0.05, **p<0.01, ***p<0.001.

To study the loss-of-function of FOXC2 in osteosarcoma cell lines, FOXC2 was silenced using lentiviral shRNA transduction in the 143B and U2OS cell lines, which have relatively high and moderate endogenous levels of FOXC2, respectively. Western blotting and qRT-PCR analyses of the FOXC2 protein and mRNA expression were used to assess the efficiency of FOXC2 silencing in cell lines transduced with FOXC2 shRNAs in comparison to the respective control cells transduced with scrambled shRNA (scr) (Figure 3A). Cell lines with the most effective knockdown of FOXC2, 143B-FOXC2 sh1 and U2OS-FOXC2 sh3, were selected for *in vitro* functional analyses. We confirmed that FOXC2 knockdown did not alter the proliferation rate or viability of these cell lines (Supplementary Figure S2). Malignant cancer is characterized by its ability to survive without support from the basement membrane, which can be mimicked *in vitro* by anchorage-independent growth in soft agar. To analyze the effect of FOXC2 downregulation on anchorage-independent growth, 143B and U2OS osteosarcoma cells with scrambled shRNA and with FOXC2-specific shRNAs were assessed for colony formation in soft agar. Downregulation of FOXC2 resulted in visibly decreased size and number of soft agar colonies in both cell lines (Figure 3B). To determine if FOXC2 contributes to invasive capability of 143B and U2OS cells, we performed the Matrigel invasion assay and evaluated the amount of cells that invaded a Matrigel after 48 hours. Our results show that downregulation of FOXC2 decreases the ability of 143B and U2OS cells to break down extracellular matrix and migrate toward the chemoattractant (Figure 3C).

### Silencing of FOXC2 expression results in decreased metastatic seeding of osteosarcoma cells *in vivo*

**Figure 4:**
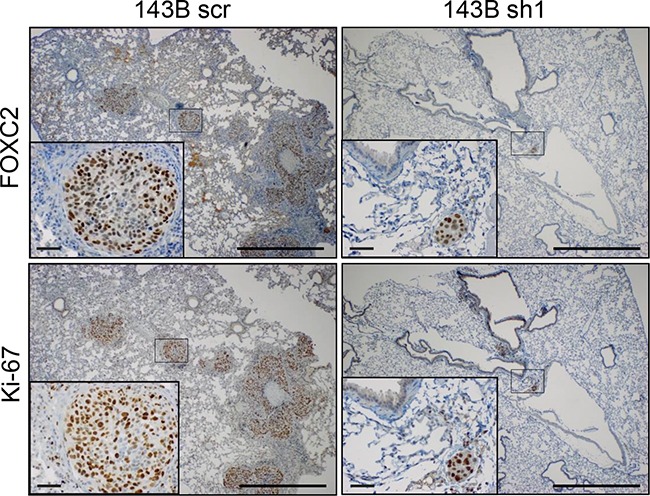
FOXC2 silencing diminishes metastatic lung colonization Mice injected with control 143B cells (143B scr) and FOXC2 knockdown 143B cells (143B sh1) via the tail vein were monitored for tumor development up to 28 days. Representative images of consecutive paraffin sections of lungs stained with FOXC2 and Ki-67 antibodies to detect tumor nodules. The insets show enlarged rectanglular areas in the main image. Main scale bar = 1 mm; Inset scale bar = 50 μm.

The highly aggressive osteosarcoma cell line 143B has been shown to metastasize to the lungs after injection of cells into nude mice [27]. Metastatic seeding *in vivo* was assessed by injecting 143B scr, 143B sh1, and 143B sh2 cells into nude mice via the tail vein. Mice were monitored for tumor development for up to 28 days. Two mice injected with 143B scr cells became moribund at days 16 and 21. Necropsy revealed that both mice had tumor nodules in the lung. At day 28, all surviving mice were euthanized and their lungs were harvested, fixed in formalin and scored for tumor nodules visible to the eye. More mice injected with 143B scr cells developed visible lung nodules (6 out of 10 mice) compared to 143B sh1 (0 out of 10 mice) and 143B sh2 (1 out of 5 mice) (Supplementary Table S2). Lungs from mice injected with 143B scr and 143B sh1 cells were examined by immunohistochemistry with antibodies against FOXC2 and the cellular marker of proliferation Ki-67. Multiple large lung nodules were observed in mice injected with 143B scr cells (Figure 4). Microscopic tumor nodules were observed in several lung sections of mice injected with 143B sh1 cells but the nodules were FOXC2 positive, indicating that they arose from cells with incomplete knockdown of FOXC2 (Figure 4). These data suggest that FOXC2 may play a critical role in osteosarcoma cell invasion and metastasis to distant organs.

### FOXC2 enhances the ability of osteosarcoma cells to propagate tumors *in vivo*

**Figure 5:**
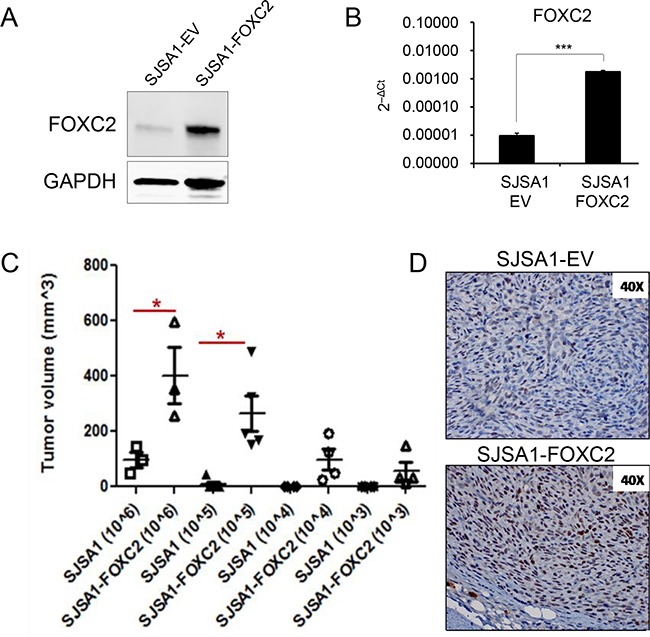
Ectopic FOXC2 enhances *in vivo* tumor-propagating ability in osteosarcoma cells **A.** Western blot and **B.** qRT-PCR analysis of FOXC2 expressionin SJSA1 cells transduced with an empty vector (SJSA-EV) or human FOXC2 (SJSA-FOXC2). **C.** Control and FOXC2-overexpressing SJSA1 cells were subcutaneously injected into 8 week-old female nude mice at limiting dilutions. Cells were injected at 4 different dilutions (10^6^, 10^5^, 10^4^ and 10^3^ cells/200 μl) and tumors were measured at 16 days post-injection. *p<0.05. **D.** Representative images of immunohistochemical staining for FOXC2 expression in paraffin sections of SJSA1-EV and SJSA1-FOXC2 tumors. ***p<0.001.

The tumorigenic potential of cancer cells is thought to be driven by a small population of tumor-propagating cells. To study the role of FOXC2 in osteosarcoma tumor propagation, we ectopically expressed FOXC2 in the SJSA1 osteosarcoma cell line, which has the lowest levels of endogenous FOXC2 compared to the U2OS and 143B cell lines (Figure 2A). SJSA1 cells were transduced with an empty vector (SJSA1-EV) or vector carrying human FOXC2 (SJSA1-FOXC2). Increased expression of the FOXC2 protein and mRNA in the SJSA1-FOXC2 cells was confirmed by Western blotting and qRT-PCR, respectively (Figure 5A, 5B). In a soft agar assay, SJSA1-FOXC2 cell colonies were visibly larger than SJSA1-EV cell colonies (data not shown). To quantitatively analyze the role of FOXC2 in tumor propagation, we injected limiting dilutions of SJSA1-EV and SJSA1-FOXC2 cells subcutaneously into nude mice and allowed for tumor development up to 16 days. Mice injected with SJSA1-FOXC2 cancer cells were able to form tumors with as low as 1×10^3^ cells, while SJSA1-EV cells injected at dilutions lower than 1×10^5^ had no detectable tumors at day 16 (Figure 5C). Additionally, at 1×10^5^ and 1×10^6^ dilutions, SJSA1-FOXC2 cells formed significantly bigger tumors than SJSA1-EV cells (Figure 5C). Immunohistochemical analysis confirmed higher levels of nuclear FOXC2 protein in the SJSA1-FOXC2 tumors in comparison to the SJSA1-EV tumors (Figure 5D). These data suggest that FOXC2 may be involved in enhancing the ability of tumor cells to propagate tumor growth *in vivo*.

### FOXC2 is required for maintenance of the molecular characteristics of tumor-propagating cancer cells

**Figure 6:**
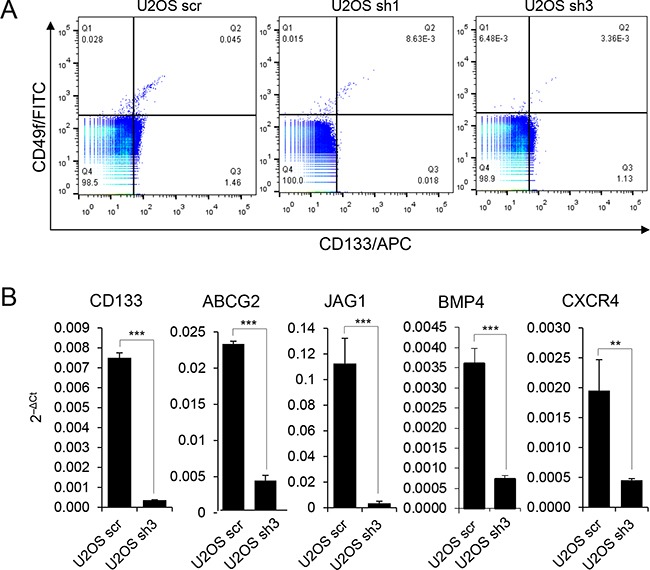
FOXC2 is required for the expression of molecular markers of tumor-propagating cells **A.** Control U2OS cells (scr) and FOXC2 knockdown U2OS cells (sh1 and sh3) were analyzed by FACS for intracellular FOXC2 and cell surface marker co-expression of CD133 and CD49f. **B.** qRT-PCR analysis of osteosarcoma tumor-propagating cell molecular markers in control U2OS cells and U2OS cells with FOXC2 knockdown. **p<0.01, ***p<0.001.

We analyzed whether FOXC2 expression is associated with expression of osteosarcoma stem cell markers CD133 and CD49f. Control U2OS cells (U2OS scr) and U2OS cells with FOXC2 knockdown (U2OS sh1 and U2OS sh3) were analyzed for co-expression of the cell surface markers CD133/CD49f (Figure 6A). FOXC2 knockdown resulted in a significant decrease in cell surface expression of CD133/CD49f stem markers (Figure 6A and Supplementary Figure S3). Next, we examined by qRT-PCR if FOXC2 knockdown in U2OS cells affects the expression of several genes known to be enriched in tumor-propagating cells, such as the cell surface marker CD133, multidrug resistance pump ABCG2, Notch signaling receptor ligands JAG1 and BMP4, and chemokine receptor CXCR4 [28–30]. We demonstrated that U2OS cells with FOXC2 knockdown (U2OS sh3) had decreased levels of CD133, ABCG2, JAG1, BMP4 and CXCR4 in comparison to U2OS scr cells (Figure 6B).

### FOXC2 effects on osteosarcoma invasion and metastasis are mediated by CXCR4

**Figure 7:**
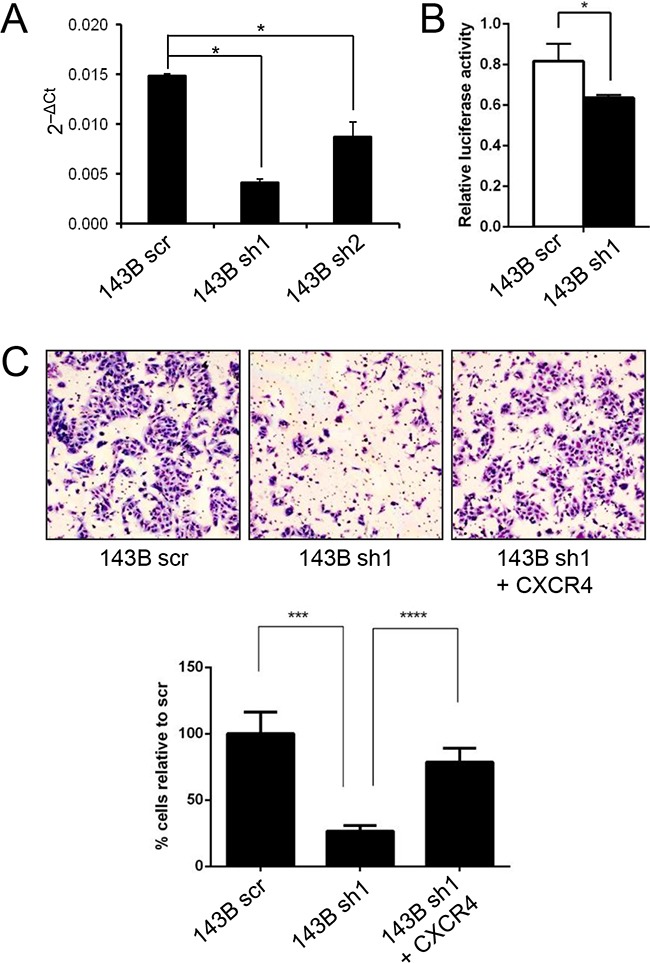
CXCR4 is a functional downstream target of FOXC2 **A.** Comparison ofqPCR levels of CXCR4 in control 143B cells (143B scr) and 143B cells with FOXC2 knockdown (143B sh1 and sh2). **B.** CXCR4-luciferase reporter assay with a 2.7-kb DNA fragment containing the human CXCR4 promoter in control 143B cells and 143B cells with FOXC2 knockdown. *p<0.05. **C.** Representative images and graphical quantifications of 12 h Matrigel invasion by control cells (143B scr), FOXC2 knockdown cells (143B sh1), and FOXC2 knockdown cells with CXCR4 overexpression (143B sh1 + CXCR4) in the presence of 10% FBS chemoattractant. ***p<0.001, ****p<0.0001.

CXCR4 has been shown to be a direct target of FOXC2 in endothelial cells [31]. We found that overexpression of FOXC2 in SJSA1 cells leads to elevated levels of CXCR4 (data not shown) and that the knockdown of FOXC2 in 143B cells leads to decreased levels of CXCR4 mRNA (Figure 7A) and decreased CXCR4-luciferase promoter activity (Figure 7B). Forced re-expression of CXCR4 in the 143B FOXC2 knockdown cells restored their invasive capabilities *in vitro* (Figure 7C) and *in vivo* (Table S2), indicating that CXCR4 is a downstream target and a functional mediator of FOXC2 signaling in osteosarcoma.

## DISCUSSION

Osteosarcoma is an aggressive pediatric tumor with poor overall prognosis despite advances in surgical techniques and combination chemotherapy regimens [2]. Chemoresistance and recurrence due to early metastatic dissemination are the major contributors to poor prognosis. Osteosarcoma is a heterogeneous malignancy consisting of phenotypically diverse cells with varying degrees of differentiation [32]. In this hierarchy, poorly-differentiated cancer cells with stem-cell characteristics, also known as tumor-propagating cells, are considered to be responsible for tumor initiation, propagation, dissemination, metastasis, recurrence, and chemotherapy resistance [6]. Targeting this cell population may provide a novel strategy in the treatment of osteosarcoma. Thus, there is great interest in key signaling pathways responsible for the maintenance of tumor-propagating cells.

In this study, we demonstrated that FOXC2 is required for many of the properties and functions of osteosarcoma tumor-propagating cells, including resistance to anoikis, increased migratory and invasive capabilities. Previous studies have shown that FOXC2 directly regulates expression of the chemokine receptor CXCR4 in endothelial cells during blood vessel formation [31]. It has also been shown that CXCR4 is expressed in osteosarcoma and may be involved in tumor lung metastases [33]. In this study, we showed that FOXC2 regulates the expression of CXCR4 in osteosarcoma. Reduced cell invasion and metastatic abilities of FOXC2 knockdown cell lines can be restored by overexpression of CXCR4, indicating that CXCR4 is a mediator of FOXC2 activity in osteosarcoma.

## MATERIALS AND METHODS

### Cell lines

The human osteosarcoma cell lines U2OS, 143B, and SJSA1 were obtained from the American Type Culture Collection (ATCC) and were propagated in DMEM, 10% FBS and 1% penicillin/streptomycin (PS) at 37°C with 5% CO_2_. Early-passage cells were used for all experiments.

### Anoikis assay

Osteosarcoma cell lines were seeded at a density of 1 × 10^6^ cells per well in poly-HEMA-coated 6-well plates. The cells were incubated in 5% CO2 at 37°C for 5 days prior to analysis. The media were changed regularly to maintain the presence of growth factors and pH. Cells were compared to input cells grown in normal adherent growth conditions at 70-80% confluency. Cell viability was analyzed by trypan blue exclusion. All cells showed over 90% viability prior to cell harvest for Western blot analysis. Each assay consisted of replicate wells and was repeated at least twice.

### Transduction of osteosarcoma cell lines with plasmids

Detailed methods for lentiviral and retroviral infection are described in our previous publication [24]. Briefly, the lentiviral plasmids pLKO.1 scrambled and pLKO.1-FOXC2 sh (Sigma) were co-transfected with the delta 8.9 packaging plasmid and envelope plasmid VSVG into Lenti-X™ 293T cells using Lipofectamine 2000 (Invitrogen) in a 10 cm^2^ dish. The retroviral vectors pWZL-blast and pWZL-FOXC2 (provided by Sendurai Mani, MD Anderson Cancer Center) were transfected into LinXA cells using Lipofectamine 2000 (Invitrogen) in a 10 cm^2^ dish. After 72 hours, viral supernatant was harvested into a 50 ml conical tube using a 0.45 μm filter syringe. Viral supernatants were concentrated (10X) using either Lenti-X concentrator (Clontech) or a Retro-X concentrator (Clontech) following the manufacturer's protocol. Cells were pelleted and resuspended in 1 ml of cell media (DMEM, 10% FBS and 1% PS). Concentrated lentivirus or retrovirus (200 μl with 8 μg/ml of polybrene) was applied to the ostesarcoma cell lines 143B (pLKO.1), U2OS (pLKO.1), and SJSA1 (pwzl), one day after seeding 3×10^4^ cells/well. After 48 hours, 143B and U2OS cells transduced with lentiviruses were selected using 10 μg/ml of puromycin while SJSA1 cells transduced with retroviruses were selected using 10 μg/ml of blasticidin. pReciever-CXCR4 (EX-Z3039-M03, GeneCopoeia, Inc.) was transfected into 143B-FOXC2 sh1 cells using Lipofectamine 2000 (Invitrogen). Cells were selected with 500 μg/ml of Geneticin.

### Immunohistochemistry

Immunohistochemical studies involving human tissue samples were approved by the Cedars-Sinai Institutional Review Board. Formalin-fixed paraffin-embedded tissue sections were deparaffinized, rehydrated and stained with antibodies against human FOXC2 (N. Miura) at 1:100 dilution and Ki-67 (Santa Cruz) at 1:400 dilution using the protocol described in our previous publication [34] except that 5% milk was used as a blocking reagent.

### cDNA synthesis and quantitative real time PCR (qRT-PCR)

RNA was isolated from osteosarcoma cell lines using the RNeasy mini kit (Qiagen) and cDNA was synthesized using the QuantiTect reverse transcription kit (Qiagen). To perform qRT-PCR, 50 ng of cDNA was added to iQSYBR-Green supermix (Bio-Rad) with primers specific for the human target gene. The qRT-PCR samples were prepared in triplicates using a 96-well format plate in an iCycler optical module thermocycler (Bio-Rad). Samples were normalized to GAPDH and 18s ribosomal RNA and the 2^−Δct^ values were determined for analysis of relative gene expression.

### Western blot analysis

Whole cell lysates were prepared using RIPA buffer (Sigma) containing a protease inhibitor cocktail (Roche) and a phosphatase inhibitor cocktail (Roche). Proteins (10-50 μg) were separated using 4-20% gradient TrisHCl gel (Bio-Rad), transferred into a nitrocellulose membrane (Bio-Rad) and incubated with antibodies against FOXC2 (N. Miura) and GAPDH (loading control) as described in our previous publication [35]. Western blots were scanned and analyzed using the Li-Cor Odyssey infrared imaging system.

### Soft agar assay

DMEM media (Cellgro) containing 0.8% sea plaque agar, 10% FBS and 1% PS were layered into a 6-well plate and incubated at room temperature overnight in a tissue culture hood. The following day, 3×10^3^ of SJSA1 and 5×10^3^ of U2OS and 143B cells were resuspended in 0.5% agar media and seeded onto the 0.8% agar. Cells were incubated at 37°C with 5% CO_2_ for 12 days and analyzed for viable colonies using iodonotrotetraxolium chloride staining (Sigma).

### Cell invasion assay

Cells were seeded at 5×10^4^ cells/well in the top chamber of a BD Matrigel invasion chamber (BD Bioscience). Media containing 10% FBS and 0% FBS were added to the bottom wells as a chemoattractant and negative control, respectively. Cells were incubated for 12-48 hours to allow for invasion through Matrigel and then analyzed by histological staining (Diff-Quik, Siemens Healthcare Diagnostics).

### Xenograft transplantation of human osteosarcoma

All animal procedures were performed in accordance with the NIH Guide for the Care and Use of Laboratory Animals and approved by the Cedars-Sinai Medical Center Institutional Animal Care and Use Committee. Eight week-old nude (Nu/Nu) mice were purchased from Charles River Laboratories. Human osteosarcoma cell lines were resuspended in PBS and 200 μl was subcutaneously injected into nude mice. Prior to injection, the mice were anesthetized with isoflurane inhalation. To analyze osteosaroma lung metastasis, 2×10^6^ 143B cancer cells in 200 μl PBS were injected into the tail vein of nude mice. Mice were euthanized by CO_2_ asphyxiation followed by cervical dislocation at the end point and their lungs were harvested and fixed in formalin for subsequent paraffin embedding and immunohistochemical staining.

### Flow cytometry analysis

The cells were washed with ice-cold phosphate-buffered saline (PBS), fixed in fixation/permeabilization buffer (eBioscience) according to the manufacturer's protocol and stained with antibodies against FOXC2 (N. Miura) or CD133 (eBioscience) and CD49f (eBioscience) or isotype controls, followed by a secondary fluorescent antibody against primary antibody isotypes (Invitrogen). The cells were washed three times with permeabilization buffer (eBioscience) and analyzed using an LSRII flow cytometer (BD Biosciences).

### Luciferase reporter assay

pGL2-CXCR4-luc reporter with a 2.7-kb DNA fragment containing the human CXCR4 promoter and control pGL2-luc reporter are gifts from Dr. Wilhelm Krek (ETH Zurich). 1 × 10^4^ cells per well were seeded in a 24-well plate and maintained in DMEM media without PS before transfecting them the following day with 100 ng pGL2 and 4 ng Renilla reporters. One day after cell transfection, Dual-Luciferase Reporter Assay (Promega) was performed according to the manufacturer's instructions. Normalization methods include normalization to Renilla luciferase activity and normalization to pGL2-luc control.

### Statistical analysis

The statistical analyses were performed using GraphPad Prism (version 6.0; GraphPad Software). The *in vitro* experiments were performed in triplicate wells and each experiment was performed at least two or three times. Data were expressed as the mean ± SEM in triplicate wells in a representative biological replicate. Intergroup differences in *in vitro* and *in vivo* assays were assessed by Student's *t*-test. Statistical significance is denoted with asterisks in the figures.

## SUPPLEMENTARY MATERIALS FIGURES AND TABLES


