# Supplementary material for: FOXC2 augments tumor propagation and metastasis in osteosarcoma
Source: Oncotarget. 2016 Sep 13;7(42):68792–802. doi: 10.18632/oncotarget.11990 (PMC5356590; doi:10.18632/oncotarget.11990)
Supplement: Supplementary file 1 [file oncotarget-07-68792-s001.pdf]

FOXC2 augments tumor propagation and metastasis in osteosarcoma

SUPPLEMENTARY FIGURES AND TABLES

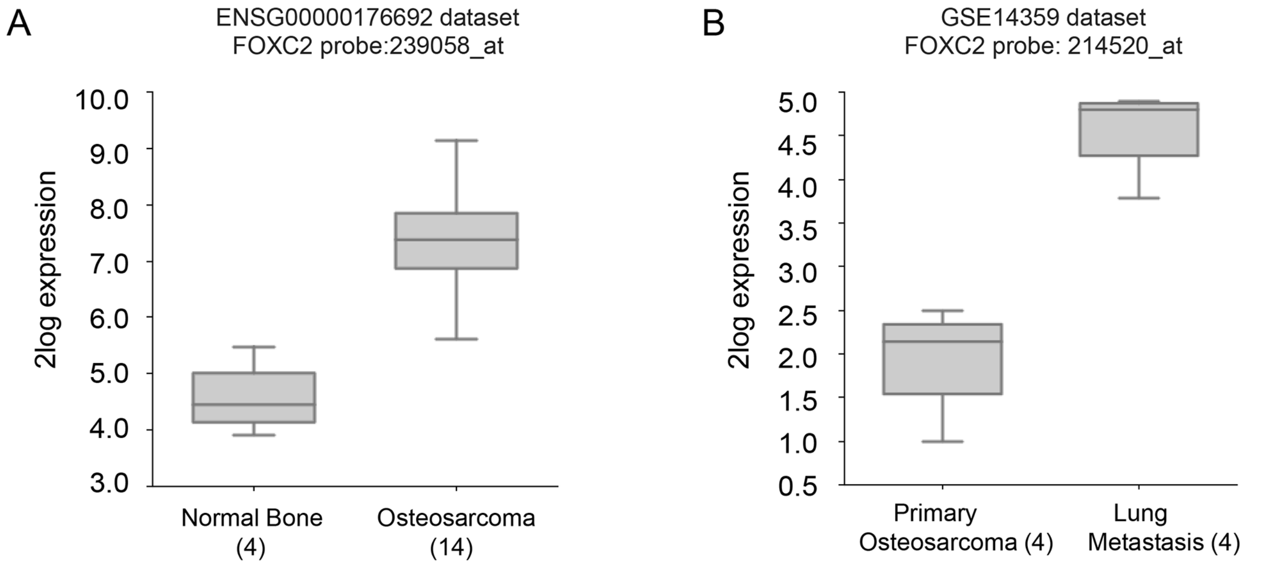

ANOVA	sum_square	df	mean_square	F	p-value
Between	24.611	1	24.611	27.126	8.6e-05
Within	14.517	16	0.907	-	-

ANOVA	sum_square	df	mean_square	F	p-value
Between	13.817	1	13.817	39.633	7.5e-04
Within	2.092	6	0.349	-	-

Supplementary Figure S1: FOXC2 mRNA is upregulated in osteosarcoma. A. Osteosarcoma samples in comparison to normal bone (Affymetrix GeneChip Human Genome U133 Plus 2.0 Array). **B.** Lung metastases in comparison to primary osteosarcoma (Affymetrix Human Genome U133A Array). The number of samples in each group is indicated in parentheses. Data were analyzed using the R2 Genomics analysis and visualization platform (http://r2.amc.nl).

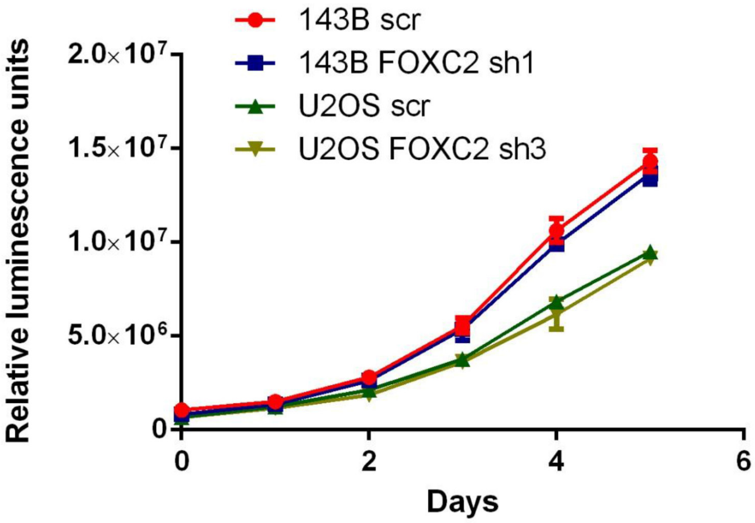

Supplementary Figure S2: FOXC2 knockdown in osteosarcoma cell lines does not affect cell proliferation/viability. Cell proliferation/viability in control cell lines (143B scr and U2OS scr) and FOXC2 knockdown cell lines (143B FOXC2 sh1 and U2OS FOXC2 sh3) was determined using the CellTiter-Glo luminescent cell viability assay. 1000 cells per well were seeded in 96-well plates. Luminescent activity was determined on days 0, 1, 2, 3, 4, and 5.

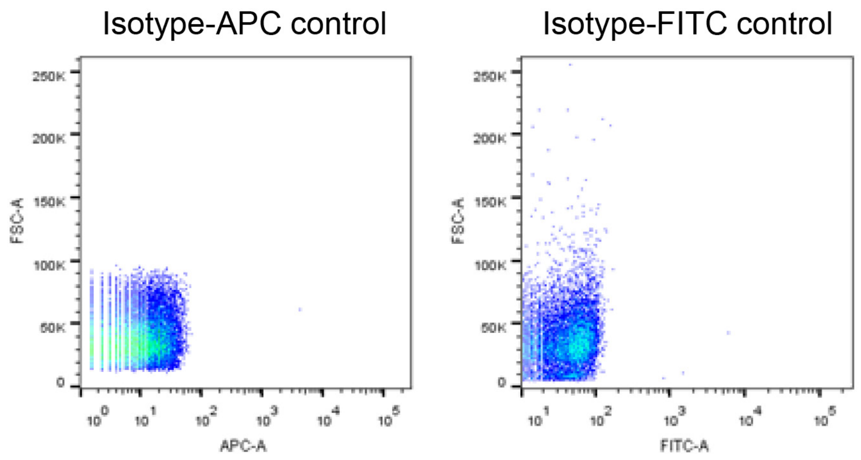

Supplementary Figure S3: Isotype controls for Figure 6A. Isotype APC and FITC controls were applied as negative controls for flow-cytometry.

Supplementary Table S1: Tumor biopsies from patients diagnosed with Ewing's sarcoma, embryonal rhabdoid sarcoma and osteosarcoma were analyzed for FOXC2 protein expression by immunohistochemistry

Tissue Sample ID	Tumor Type	FOXC2
S1	Ewing's sarcoma	neg
S2	Ewing's sarcoma	neg
S3	Ewing's sarcoma	neg
S4	Ewing's sarcoma	neg
S5	Ewing's sarcoma	pos
S6	Ewing's sarcoma	pos
S7	Ewing's sarcoma	pos/neg
S8	Embryonal rhabdoid	neg
S9	Embryonal rhabdoid	pos
S10	Osteosarcoma (lung met)	pos
S11	Osteosarcoma (lung met)	pos
S12	Osteosarcoma (lung met)	pos
S13	Osteosarcoma (lung met)	pos
S14	Osteosarcoma	pos
S15	Osteosarcoma	neg
S16	Osteosarcoma	pos/neg
S17	Osteosarcoma	pos/neg
S18	Osteosarcoma	pos
S19	Osteosarcoma	pos

The slides were scored for the presence or absence of nuclear FOXC2 staining as positive (2+ or 3+), positive/negative (1+), and negative (no stained tumor cells).

Supplementary Table S2: Mice injected with control 143B scr cells, FOXC2 knockdown 143B cells (143B sh1 and 143B sh2) and FOXC2 knockdown 143B cells with CXCR4 overexpression (143B sh1 + CXCR4) via the tail vein were monitored for tumor development up to 28 days

143B cell type	Mice with lung metastases
143B scr	6/10
143B sh1	0/10
143B sh2	1/5
143B sh1 + CXCR4	9/9

At day 28, lung tissues were harvested and scored for the presence of metastatic nodules visible to the eye.
